# Retraction: Structural Basis of the Substrate Specificity and Enzyme Catalysis of a *Papaver somniferum* Tyrosine Decarboxylase

**DOI:** 10.3389/fmolb.2017.00069

**Published:** 2017-09-27

**Authors:** Frontiers Editorial Office

The Journal and the authors retract the 9 February 2017 article cited above.

Following article publication, the authors reported that the data upon which the study was based is inconsistent with their own archived data files. Since this discrepancy cannot be resolved by the authors, the authors request that the paper be retracted.

The retraction of the article was approved by the Field Chief Editor of Frontiers in Molecular Biosciences.

